# Quality of Service Class Identifier (QCI) radio resource allocation algorithm for LTE downlink

**DOI:** 10.1371/journal.pone.0210310

**Published:** 2019-01-25

**Authors:** Maharazu Mamman, Zurina Mohd Hanapi, Azizol Abdullah, Abdullah Muhammed

**Affiliations:** Department of Communication Technology and Networking, Faculty of Computer Science and Information Technology, University Putra Malaysia, UPM Serdang, Selangor, Malaysia; Southwest University, CHINA

## Abstract

The increasing demand for network applications, such as teleconferencing, multimedia messaging and mobile TV, which have diverse requirements, has resulted in the introduction of Long Term Evolution (LTE) by the Third Generation Partnership Project (3GPP). LTE networks implement resource allocation algorithms to distribute radio resource to satisfy the bandwidth and delay requirements of users. However, the scheduling algorithm problem of distributing radio resources to users is not well defined in the LTE standard and thus considerably affects transmission order. Furthermore, the existing radio resource algorithm suffers from performance degradation under prioritised conditions because of the minimum data rate used to determine the transmission order. In this work, a novel downlink resource allocation algorithm that uses quality of service (QoS) requirements and channel conditions to address performance degradation is proposed. The new algorithm is formulated as an optimisation problem where network resources are allocated according to users’ priority, whereas the scheduling algorithm decides on the basis of users’ channel status to satisfy the demands of QoS. Simulation is used to evaluate the performance of the proposed algorithm, and results demonstrate that it performs better than do all other algorithms according to the measured metrics.

## 1 Introduction

In the past decades, considerable development has been observed in communication networks. Early mobile communication systems offered video services, data and voice. With the emergence of advanced communication devices, networks now support such services as video streaming, web browsing and online gaming. These services, which normally have different delay constraints, bandwidth requirements and quality of service (QoS) requirements, cause network problems. The Third Generation Partnership Project (3GPP) introduced Long Term Evolution (LTE) as an answer to the above challenges [1]. LTE aims to support peak data rate, high spectral efficiency, high coverage area and improved latency. To achieve high peak data rate, LTE adopts orthogonal frequency division multiple access (OFDMA) as the downlink access technology and exploits single-carrier frequency division multiple access for uplink transmission. Furthermore, in only a decade, the amount of data handled by LTE networks has increased by a factor of 100; from under 3 exabytes in 2010, it is expected to exceed 190 and 500 exabytes by 2018 and 2020, respectively [2].

Despite the benefits of LTE, the resource allocation issue remains a significant problem. Allocation of resources to numerous users with different QoS requirements is challenging. Radio resource allocation is needed to provide QoS requirements to many users. Radio resource allocations have been extensively studied in OFDMA systems using packet schedulers. These schedulers are responsible for selecting good frequency and time resolutions, which are used in the allocation of resource blocks (RB) between different user equipment (UE) with consideration for channel conditions and QoS requirements; thus, packet schedulers play an important role. QoS requirements should be met for each bearer. A bearer is established amongst UE and packet data network gateway to show the use of data flow in the evolved packet system. Each bearer has a corresponding QoS class identifier (QCI), and each QoS is categorised with the service type, priority, packet error rate (PER) and packet delay budget (PDB) [3].

To effectively support the present types of services, effective use of scarce shared spectrum resources is necessary [1]. The goal of efficient scheduling approach is critical in meeting LTE targets because choosing a suitable scheduling mechanism is not well defined in the 3GPP specifications for LTE, but vendors are free to adopt, configure and implement their own algorithms depending on the problems of the system [4]. Nevertheless, achieving all the intended objectives simultaneously is difficult [5]. Each problem solved can lead to additional ones. For instance, radio resource algorithms intended to maximise system throughput are not appropriate for handling guaranteed bit rate traffic [6]. Hence, the major problem is developing a scheduling mechanism which creates a trade-off between the system performances.

Therefore, this research proposes a new radio resource algorithm for downlink LTE networks by considering QoS requirements for each service type. The algorithm explains how to meet the demand of bearer traffic by allocating the available bandwidth such that the bearer traffic and bandwidth requirements in the network are achieved without sacrificing total system throughput. The algorithm is formulated as a radio resource optimisation problem which provides a fast and precise solution to determine the closely optimal resource allocation decisions. The relative advantages of many bearers demanding resource allocation are measured by using a class range to the bearers. This novel scheduling algorithm mechanism is applied in time domain scheduling, thus improving system performance efficiency. Five measures are used to evaluate the performance of the proposed algorithm, namely, total sector throughput, average user throughput, fairness, packet loss ratio (PLR) and delay.

The remainder of this paper is organised as follows. Section 2 provides the background of the LTE networks and cites several related works. Section 3 presents the model for the proposed algorithm and its details. Section 4 describes the simulations results. Section 5 provides the conclusions and recommendations for future directions.

## 2 Related works

In this part, three issues are considered and discussed, namely, scheduler in downlink LTE network, LTE performance guarantee and radio resource allocation optimisation. In addition, state-of-the-art works in LTE are reviewed to offer effective, reliable and optimised downlink scheduling algorithms between bearers of various types in LTE networks.

### 2.1 Scheduler in downlink LTE network

The packet scheduler located at the enodeB manages and schedules packets according to their service types. When many packets are waiting to be served, the scheduler should be able to arrange them according to service order, transmission rate and service time to guarantee satisfying their QoS requirement. The scheduler at the media access control (MAC) layer plays a vital role in measuring network performance. It allocates bandwidth to UE and is liable for determining how downlink and uplink channels are used by the enodeB and the UEs of a system [7].

**Fig 1:**
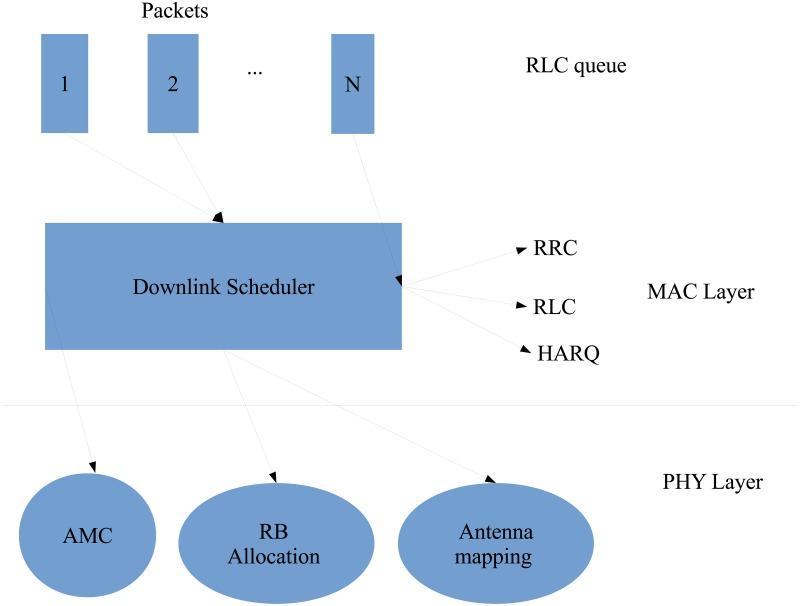
Scheduler in LTE protocol stack.

**Fig 2:**
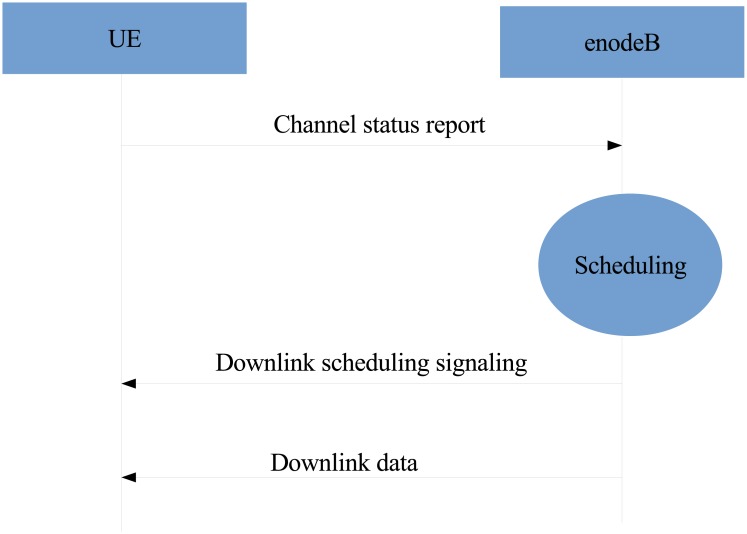
Downlink scheduling process.

Fig 1 illustrates the scheduler in the LTE protocol stack which aims to determine how to distribute radio resources to users [7]. The protocol stack functions comprise radio link control (RLC), radio resource control (RRC) and hybrid automatic repeat-request (HARQ). The listed functionalities are controlled by a downlink scheduler at the MAC layer. The RLC layer is located between RRC and HARQ. It can communicate with RRC using a service access point and logical channels with HARQ. This layer can reformat the packet data units into a size which can be accommodated by the MAC layer. Additionally, the RLC reorders the packet data unit when it is received out of order because of the HARQ operation executed in the MAC layer. Fig 2 shows how the scheduling process takes place at the scheduler by considering the channel status. The enodeB obtains the channel status from the feedback received from the users’ channel quality indicator (CQI). Resources are assigned to users on the basis of QoS requirement, fairness and channel status. The enodeB sends data and scheduling signalling using the scheduling results. Scheduled users receive data in light of RB allocation results, antenna selection and MCS in the downlink scheduling signalling.

To achieve scheduling performance targets, the algorithm must be implemented at the enodeB located at the MAC layer [8]. The enodeB allocates a portion of network bandwidth to be shared among users using many approaches. LTE employs OFDMA as an approach to accommodating UEs with the different type of QoS requirements. Each OFDMA frame consists of 10 transmission time interval (TTIs). Each TTI can pan over 0.5 ms, which is equivalent to seven OFDM symbols in cyclic prefix and normal form configurations. The available system bandwidth is divided into sub-channels. Each sub-channel is of 180 kHz frequency and consists of 12 consecutive OFDM sub-carriers with equivalent spaces [9].

To reduce complexity and increase design flexibility, recent work focuses on a two-level framework [10]–[11]. This framework divides the radio resource technique into frequency domain (FD) and time domain (TD) schedulers working independently of each other. The TD scheduler chooses a group user request to be scheduled in the next TTI according to their QoS requirements. The FD scheduler often receives the selected group user and determines the RBs which should be allocated to each group user by using channel quality. The proposed algorithm in this work emphasises TD to provide effective utilisation of resources between users. Moreover, FD can be combined with most schedulers in the previous works to accomplish the provisioning of spectrum utilisation effectively.

### 2.2 Performance guarantee

In 4G cellular networks, performance guarantee means the manner by which network utilisation is efficiently expressed in term of fairness, QoS and system throughput provisioning. QoS illustrates how effectively and dependably a network can attain a certain level for real-time (RT) and non-RT (NRT) traffic. Several legacy approaches have been proposed for wireless communication networks for evaluating the performance of QoS requirements, as shown in Eqs (1)–(5), but each has its own drawback. For ease of reference, some notations used in this paper are listed in Table 1.

**Table 1:** Some notations used in this paper.

Notation	Definition
Ri (t)	Instantaneous data rate experienced by user i
k	Number of resource block
t	Current time
*T*_*i*_	Last time a user was served
*λi*(*t*)	Average data rate for user i
*Nrt*	The number of active downlink RT flows
*αiDHOL*, *i*	Delay of the first packet to be transmitted by the *ith* user
$d_{k}^{i}\left(t\right)$	Expected data-rate for the *ith* user at a time t on the *kth* RB
*R*^−*i*^	Past average throughput achieved by the *ith* user
*D*_*i*_	The HOL packet delay of *user*_*i*_
*r*_*i*_	Bit data rate
$R_{i}^{\left(c\right)}$	Code rate associated with the MCS
*M*_*i*_	Size of the MCS
*L*_*e*_ (*j*)	The number of data-carrying subcarriers
*n*	Maximum CQI on all RBs of the *kth* user
*r*_*k*_	Achievable data rate for *kth* user in one subframe
*R*_*k*_	Rate requirement for user k;
R	Large positive real number.

Maximum signal interference-plus-noise-ratio (SINR) algorithm aims to maximise the throughput of the enodeB signal-to-noise ratio. In this algorithm, RBs are allocated only to UEs in good channel states, whereas UEs in bad channel states will never be given RBs [12]. However, maximum SINR algorithm utilises system resources but ignores fairness. It mathematically schedules user *ith* as follows:

$$\begin{array}{c} M_{i,k}^{MSINR}=argMax_{i}\left(Ri\left(t\right)\right) \end{array}$$ (1)

where Ri(t) is the instantaneous data rate experienced by user i and k represents the number of RBs.

Round robin (RR) algorithm runs in a fixed round where active users are determined, and each user is assigned with equivalent and fixed number of time which is slotted in a cyclic manner. Users in RR are treated without any priority; hence, it is starvation-free [13]. This can be expressed mathematically as follows:

$$\begin{array}{c} M_{i,k}^{RR}=t-T_{i} \end{array}$$ (2)

where t represents the current time, *T*_*i*_ denotes the last time a user was served and *ith* is the user on the *kth* RB.

The proportional fair (PF) algorithm was proposed to address the problems of fairness and user throughput by providing a balance between them [14]. It keeps records of average throughput for all the users among the active users in the system. For a given number of time slots, the algorithm selects and transmits users with the maximum rate and prevents starvation through the weighted factor such that users under bad channel conditions can be served within a time frame. Moreover, the PF algorithm does not consider delay-sensitive users; therefore, it is insufficient for supporting RT application with superior QoS. This algorithm can be expressed in LTE behaviour as follows:

$$\begin{array}{c} M_{i,k}^{PF}=argMax_{i}(\frac{Ri(t)}{\lambda i}) \end{array}$$ (3)

where Ri(t) is defined in Eq (1) and *λi*(*t*) is the average data rate for user i.

Exponential PF rule (EXP-PF) is an algorithm developed to handle multimedia applications which can support systems in adaptive coding and modulation or time multiplexing systems. EXP-PF algorithm classifies users into best effort data services and streaming services. The best effort services use PF rule to maximise system throughput whilst fairness is achieved. Meanwhile, the streaming services using EXP rule aims to guarantee delay bound whilst fairness is maintained [4]. However, the algorithm cannot guarantee resource control “delay requirement” between RT and non-RT service users, and it maximises the throughput of the system. The algorithm can be expressed mathematically as follows:

$$\begin{array}{c} M_{i,k}^{EXP-PF}=\exp(\frac{\alpha iDHOL,i-x}{1+\surd x})\times \frac{d_{k}^{i}\left(t\right)}{R^{-i}} \end{array}$$ (4)

where $\text{x}=\frac{1}{Nrt}\sum_{i=1}^{Nrt}$ is the number of active downlink RT flows, *Nrt* denotes the number of active downlink RT flows, *αiDHOL*, *i* denotes delay of the first packet to be transmitted by the *ith* user, $d_{k}^{i}\left(t\right)$ represents expected data-rate for the *ith* user at a time t on the *kth* RB and *R*^−*i*^ denotes past average throughput achieved by the *ith* user.

Modified largest weighted delay first (MLWDF) is a sort of algorithm designed for the purpose of supporting multiple RT data users with diverse QoS requirements [15]. MLWDF provides priority to RT users. Users are served on the basis of the head of line (HOL) packet delay. It saves the probability of delayed packets above the discard bound under the highest permitted service data unit. This algorithm achieves low PLR with QoS requirement because it combines HOL packet delay with proportional fair properties when deciding on user priority. However, this algorithm is unfair because it offers a better QoS to users under favourable propagation situations than to those under other conditions. The following equation describes the metric used to represent MLWDF algorithm:

$$\begin{array}{c} M_{i,k}^{MLWDF}=argMax_{i}(\alpha_{i}\times \frac{R_{i}\left(t\right)}{\lambda_{i}\left(t\right)}\times D_{i}) \end{array}$$ (5)

where *a*_*i*_ depends on *α*_*i*_ proportionally, *D*_*i*_ refers to the HOL packet delay of user i, *R*_*i*_(*t*) and *λ*_*i*_(*t*) is as defined in Eq (3)

Many researchers also developed their own radio resource scheduling algorithms to support RT services or provide fair transmissions among flows in LTE networks. The work in [10], proposed a scheduling framework for downlink LTE to satisfy QoS requirements defined by 3GPP specifications. The algorithm employs a channel-aware service concept to prioritise guaranteed bit rate (GBR) and non-GBR bearers from diverse QCIs using a sigmoid function. PDB is achieved by GBR bearers, whereas non-GBR bearers provide high spectral efficiency. In [16], an optimal and suboptimal scheduler was proposed to improve system performance. To maximise system performance, the scheduler assigns a scheduling block (SB) to the user for a period of 1 ms, which is equivalent to the duration of TTI. However, this scheduler does not show fairness among users by providing the same duration for SB and TTI as 1 ms.

A fair downlink scheduling algorithm for 3GPP networks was proposed in [8]. For each TTI, the algorithm uses an assignment model for the distribution of resources. The algorithm creates a balance between user throughput and fairness. A delay scheduler coupled throughput—fairness resource allocation algorithm was proposed in [17]. The algorithm categorises traffic into urgent and non-urgent ones. Using an optimisation technique, this algorithm finds the trade-off between RT, QoS and NRT throughput. It operates in two phases. In the first phase, it determines the scheduling priorities for the urgent packets which guarantee QoS; the second phase aims to provide fairness in terms of channel utilisation to the non-urgent packets. The work in [13], presented a comprehensive survey of the key design issues for resource allocation for downlink scheduling, although some of the assumptions in the work are also applicable for uplink scheduling. The theoretical exercises were covered, but the implementation was ignored in RT systems owing to difficulty and high cost. Moreover, only PF strategy was used for testing the algorithm.

A service-differentiated downlink flow scheduling (S-DFS) algorithm was proposed in [18] to avoid starvation of non-GBR flows and guarantee QoS of GBR flows considering channel quality, HOL packet delay, queue length and QCI. S-DFS employs channel quality and QCI to allow flow to bid for physical RB (PRB) to guarantee QoS to GBR flows. The algorithm uses adjustable *α* and *β* to prevent some flows from occupying a large number of PRBs. In addition, it reallocates unused PRBs to flow with high demand. However, a different load scenario is ignored in this algorithm. The work in [19] proposed a scheduling algorithm for multimedia applications. This allocates resources to RT traffic because they have a high demand for network resources using delay priority function. Traffic whose delay is near the threshold value is transmitted first. However, the algorithm achieves optimum QoS performance for RT applications whilst ignoring NRT applications.

The authors in [20] proposed a multi-level queue algorithm to optimise the throughput of the network. It divides users into multiple queues on the basis of their channel states. The users in good channel states are assigned high-priority queues, whereas those in bad channel states are allocated low-priority queues. This process improves the system throughput but ignores QoS requirements. In [21], an optimal priority-based rate-guaranteed radio resource allocation for LTE downlinks was proposed. It categorises users into priority and non-priority users. Priority users have the highest priority. Hence, resources are allocated to them firstly. The remaining resources, if any, are allocated to non-priority users. However, the algorithm uses the minimum data rate for allocating resources whilst ignoring users’ QoS requirements, thereby resulting in a decrease in system performance.

The above-mentioned approaches do not achieve all the QoS requirements for diverse classes of QoS, which have a significant role for the end user. Accordingly, an optimal and priority algorithm was proposed in [21]; using minimum data rate to allocate resources to the user. However, this scheme does not account for QoS requirements for different users which lead to performance degradation. Motivated by the above, a novel radio resource scheduling algorithm is proposed, which improve the performance degradation and guarantees QoS provisioning.

### 2.3 Radio resource allocation optimisation

The downlink packet scheduler must guarantee the fulfilment of the QoS requirements of cellular 4G networks and be robust to variations in channel states. Therefore, downlink scheduling in LTE networks is an NP-hard problem because the optimised scheduling process is complex and time consuming [22]. Recently, an optimal heuristic approach for multi-users in downlink LTE was proposed. The algorithm aims to maximise the total throughput of users in the network using overbooked scenarios. However, computational complexity is high [23]. A two-level scheduling algorithm which uses cooperative game theory and a virtual token mechanism was suggested in [24]. To improve the radio resource scheduling algorithm in the downlink system, the algorithm adopts a bankruptcy and the Shapley value for efficient and fair distribution of bandwidth between flow classes. However, computational complexity and weak scalability are main concerns of this approach.

Chance-constrained programming for cognitive OFDM networks was investigated in [25]. The scheme aims to maximise spectral efficiency; however, it causes interference to primary users owing to the high transmission power. In [26], a novel scheduling algorithm based on game theory and multi-criteria decision making in LTE networks was presented. The scheme improves resource allocation for smart grid applications using bankruptcy and Shapley (cooperative game theory) techniques for order performance by similarity to ideal solution. The measure drawback of this scheme is its complexity, where scheduling decision-making consumes 1 ms.

In [21], an optimal priority algorithm (hereafter called the Benchwork algorithm) was implemented for LTE downlink scheduling. In this scheme, the scheduling problem is formulated using minimum data rate and users are served on the basis of priority order. However, selecting users without considering the class range does not yield optimal value. Additionally, other LTE performance targets must be identified to achieve efficient radio resource allocation. The major contributions of this work are the introduction of a novel radio resource scheduling algorithm and provision of an optimal solution for the proposed scheme.

## 3 Downlink radio resource allocation optimal model

To optimise numerous objectives of PF and maximise the total sector throughput subject to satisfying QoS constraints on priority, delay, user data rate and packet loss, the scheduling scheme presented here is a bearer class QoS control scheme. A bearer is a logical channel which establishes a connection between UE and enodeB. Occasionally, a user may request many services having diverse QoS requirements at a time. Therefore, to distinguish between these different services, 3GPP defined the set of characteristics for nine QCIs, as presented in Table 2.

**Table 2:** Standardized QCI [15].

QCI	Service Type	Priority	PDB(ms)	PER	Examples service
1	GBR	2	100	10^−2^	Conversational voice (VoIP)
2	GBR	4	150	10^−3^	Conversational video (live streaming)
3	GBR	5	300	10^−6^	Non-Conversational video (buffered streaming)
4	GBR	3	50	10^−3^	Real-time gaming
5	Non-GBR	1	100	10^−6^	IMS signaling
6	Non-GBR	7	100	10^−3^	Voice, Video (live streaming), interactive gaming
7	Non-GBR	6	300	10^−6^	Video streaming(buffered streaming)
8	Non-GBR	8	300	10^−6^	TCP based (e.g. www, email), chat, FTP, p2p file sharing
9	Non-GBR	9	300	10^−6^	TCP based (e.g. www, email), chat, FTP, p2p file sharing

### 3.1 Problem formulation

Every QCI has a specific packet despatching behaviour that is based on the certain specification. To fulfil the QoS class-based constraints and maximise total throughput, the class bearers are allocated to a particular QCI that is based on scheduling approach while satisfying the QoS requirements. In this proposed work, it is assume that the network model has an enodeB which allocates RBs to the k number of users. Each OFDMA has 1 ms duration and contains 12 or 14 RBs of OFDM symbols. The bit data rate *r*_*i*_ equivalent to an allocated single RB can be expressed as follows:

$$\begin{array}{c} r_{i}=\frac{R_{i}^{\left(c\right)}\text{log}2\left(M_{i}\right)}{T_{s}N_{RB}}\overset{N_{RB}}{\underset{j=1}{\sum }}L_{e}\left(j\right) \end{array}$$ (6)

where $R_{i}^{\left(c\right)}$ represents the code rate associated with the MCS, *M*_*i*_ denotes the size of the MCS, *T*_*s*_ represents the OFDM symbol duration, *N*_*RB*_ denotes the number of OFDM symbols in an RB (usually 12 or 14, according to the cyclic prefix or normal cyclic used) and *L*_*e*_(*j*) represents the number of data-carrying subcarriers.

In the LTE setup, CQI is described on the basis of channel coding rate and MCS. The scheduler will determine which MCS should be imposed if the *nth* RB is assigned to the *kth* user. Moreover, the maximum CQI on all RBs of the *kth* user can be defined as follows:

$$\begin{array}{c} n=argmax(CQI_{k,n}) \end{array}$$ (7)

where *n* represents the maximum CQI, *CQI*_*k*, *n*_ denotes the CQI of user *k* in the nth RBs, the user’s CQI on N RBs can be defined as *CQI*_*k*, *n*_ = (*CQI*_*k*,1_, *CQI*_*k*,2_,…, *CQI*_*k*, *N*_), *N* represent CQI values, *argmax* is selection in the range [*min*(*CQI*_*k*_),…, *max*(*CQI*_*k*_)].

Furthermore, let *CQI*_*k*, *n*_, n = 1, 2, …, *N*_*i*_ be a real scalar or vector sent back by the user k to show the combined channel qualities of all the subcarriers within the *nth* reported RB. Let *q*_*i*, *max*_ ∈ (1, 2, …, I) be the index of the highest-rated MCS which can be achieved by the k user for the *nth* RB at CQI value *CQI*_*k*, *n*_ i.e $q_{i,\text{max}}\left(CQI_{k,n}\right)=\;\text{argmax}\;R_{i}^{\left(c\right)}\text{log}2\left(M_{i}\right)|CQI_{k,n}$. Define *ω*_*k*, *n*_ as the resource allocation indicator for user k on the *nth* RB. Assume that each RB is allocated particularly to one user, i.e., if *ω*_*k*, *n*_ = 1 and *ω*_*k*′, *n*_ = 0 ∀ *k*′ ≠ *k*. We also assume that *C*_*k*, *j*_ represents the selection of the MCS of user k on all the RBs assigned to it in one subframe. If *C*_*k*, *j*_ = 1 then *C*_*k*, *j*_ = 0 ∀ *i* ≠ *j*. Hence, the *kth* user’s achievable data rate (bit/s) *r*_*k*_ in one subframe can be described as follows:

$$\begin{array}{c} r_{k}=\sum_{n=1}^{N}\omega_{k,n}\sum_{j=1}^{q_{i,max}\left(CQI_{k,n}\right)}C_{k,j}r^{\left(j\right)} \end{array}$$ (8)

Therefore, the proposed algorithm, which intends to attain maximum sector throughput by considering users’ achievable data rate of each user, can be expressed as follows:

$$\begin{array}{c} \max_{\omega_{k,n},C_{k,j}}\sum_{k=1}^{K}\sum_{n=1}^{N}\omega_{k,n}\sum_{j=1}^{q_{i,max}\left(CQI_{k,n}\right)}C_{k,j}r^{\left(j\right)} \end{array}$$ (9)

Subject to:

$$\begin{array}{c} r_{k}\geq R_{k} \end{array}$$ (10)

$$\begin{array}{c} \forall i\in N,k\in K:pdb_{i,k}<PDB_{k}, \end{array}$$ (11)

$$\begin{array}{c} \forall i\in N,k\in K:per_{i,k}<PER_{k}, \end{array}$$ (12)

where *R*_*k*_ denotes the rate requirement for user k, i ∈ N = {1,…, *n*} represents the scheduling bearer index selected for user i. Each bearer i is allocated to a single QCI type with class {1,…,9}. *PDB*_*k*_ and *PER*_*k*_ denote the standardised PDB and PER threshold values of the equivalent QCI type, respectively, and *pdb*_*i*, *k*_ and *per*_*i*, *k*_ represents the measured PDB and PER for bearer i from QCI type k, respectively. Eq (9) denotes the objective function defining the total data rate achieved in the present TTI. Eq (10) represents the data rates needed by users to guarantee QoS requirements. Eqs (11) and (12) illustrate the computed values for PDB and PER for bearer i.

Eq (13) ensures that each RB is allocated to a single user, whereas Eq (14) sets the limitation that all RBs for a given user must use the same MCS.

$$\begin{array}{c} if\omega_{k,n}=1,then\;\omega_{k^{\prime },n}=0,\forall k^{\prime }\neq k \end{array}$$ (13)

$$\begin{array}{c} \sum_{j=1}^{q_{i,max}\left(CQI_{k,n}\right)}C_{k,j} \end{array}$$ (14)

The problem in Eq (9) is nonlinear because of the product *ω*_*k*, *n*_, *C*_*k*, *j*_. Although we can have a solution for the optimisation approaches, global optimality cannot be assured [27]. Therefore, to prevent this predicament, the problem in Eq (9) can be converted into an equivalent linear problem by introducing an auxiliary variable *a*_*n*, *k*, *j*_ = *ω*_*k*, *n*_, *C*_*k*, *j*_.

From Eq (13) *ω*_*k*, *n*_ = 1, therefore *C*_*k*, *j*_ × 1 = *C*_*k*, *j*_. By substituting *ω*_*k*, *n*_, *C*_*k*, *j*_ with *a*_*n*, *k*, *j*_ as given in Eq (15), the problem can be linearized as illustrated below:

$$\begin{array}{c} \max_{\omega_{k,n},C_{k,j},a_{n,k,j}}\sum_{k=1}^{K}\sum_{n=1}^{N}\sum_{j=1}^{q_{i,max}\left(CQI_{k,n}\right)}a_{n,k,j}r^{\left(j\right)} \end{array}$$ (15)

Subject to (10), (11), and (12)

$$\begin{array}{c} \begin{array}{c} a_{n,k,j}\leq C_{k,j},\\ a_{n,k,j}\leq \omega_{k,n}R,\\ a_{n,k,j}\geq C_{k,j}-\left(1-\omega_{k,n}\right)R. \end{array} \end{array}$$ (16)

where R denotes a large positive real number.

Consequently, Eq (15) can be solved by the integer linear programming method [27]. Therefore, the scheduling problem for this formulated optimisation technique is the manner by which enodeB decides which user should be scheduled for each RB because users have different QoS requirements. Thus, the advantages of our proposed algorithm are maximisation of system throughput and minimisation of delay and PLR, as given in Eqs (9)–(16). Moreover, Eq (15) is an integer optimisation problem which is rendered NP-hard by the numbers of constraints and variables. Consequently, a new scheduling algorithm is proposed to minimise the complexity problem of Eq (15). Algorithm 1 further shows the pseudocode for the proposed QCI radio resource allocation algorithm for LTE downlinks.

**Algorithm 1:** Quality of Service Class Identifier (QCI) Radio Resource Allocation Algorithm for LTE Downlink

**1 Input:**

**2** K ← number of user;

**3** *N*_*RB*_← number of RBs (25);

**4** *R*_*k*_← rate requirement for user k;

**5** G ← feedback CQIs matrix on RB by user k;

**6** *P*_*k*_← priority index for user k (1 or 0);

**7** W ← available number of RBs when every user was served;

**8** *N*_*k*_← estimated number of RBs required by each user;

**9** *β*_*CQI*_← channel quality indicator;

**10** *r*_*r*_← achievable data rate;

**11** *RA*_*p*_← allocate RBs to priority users;

**12** *RA*_*np*_← allocate RBs to non-priority users;

**13** *g*_*k*_← average channel gain

**14 Initialization:**

**15** *T*_*k*_ = {}, k ∈ {1, 2, 3, …, *K*};

***16** ω*_*k*, *n*_ = 0, k ∈ {1, 2, 3, …, *K*}, n ∈ {1, 2, 3, …, *N*_*RB*_};

**17** *r*_*k*_ = 0, k ∈ {1, 2, 3, …, *K*};

**18** Calculate *g*_*k*_ = $\frac{\sum_{i=1}^{\beta_{CQI}}g_{k}}{N_{RB}}$

**19** Calculate *N*_*k*_ = $N_{RB}\times (\frac{R_{k}}{g_{k}})$

**20** Users are arranged in decreasing order of their data rate requirement

**21 for** *k = 1* to *K* **do**

**22** **if** *P*_*k*_ = 1 **then**

**23**  *RA*_*p*_ hold;

**24** **end**

**25** **if** *N*_*k*_ ≤ *W* **then**

**26**  allocate *N*_*k*_ to user k, and calculate *r*_*k*_ as in Eq (8)

**27** **end**

**28** select the *N*_*k*_ RBs of user k based on Eq (7), and insert RBs in the set *T*_*k*_

**29** RBs in the set *T*_*k*_ are aranged based on smallest CQI

**30** **if** *r*_*r*_ ≥ *R*_*k*_ **then**

**31**  k = k + 1

**32** **end**

**33** **if** *P*_*k*_ = 0 **then**

**34**  *RA*_*np*_ hold;

**35** **end**

**36** if RBs still remains after allocation to non-priority users, then redistribute equally to priority users

**37 end**

### 3.2 Complexity analysis

In this section, the complexity analysis for PF, EXP-PF, MLWDF and the proposed algorithm is given by considering allocation and computational times [7]. The complexity of PF algorithm is calculated by the selection of the best metric for user n, and its scheduling complexity is given as O(log n) for each RB. MLWDF algorithm adds a weighted factor and HOL packet delay to the PF metric. However, its computational time differs from that of PF, and it chooses from several users. Hence, it has an allocation time complexity of O(log n) for each RB. Similarly, for EXP-PF algorithm, an exponential delay parameter for calculating priority metrics is computed by focusing on resource allocation cost, thereby resulting in the allocation complexity of O(log n). Furthermore, the scheduling complexity for the proposed algorithm is based on a sorting technique. Based on simple insertion sorting, it has the complexity of *n*^2^, whereas for quick, merge and heap sorts, the complexity is O(log n).

Similarly, in terms of computational time, the proposed algorithm shows how time is minimised efficiently, and the computational time is calculated on the basis of packet loss by considering the initial and final times of transmission. Hence, the time complexity based on this scenario is O(1). Furthermore, assume K users with different services types. Thus, in every TTI, the time to count K users with I number of service generated is *O*(K*I). In this algorithm, we consider two cases for resource allocation, i.e. channel condition and QoS requirement of user traffic, which are implemented only once in the process for (2^*K*^−1) hence resulting in the time complexity of *O*(I*(2^*K*^−1). Therefore, the proposed algorithm’s computational time is computed as *O*(K*I)+*O*(I*(2^*K*^−1).

## 4 Simulation results and discussion

In this research, the performance of the proposed algorithm is compared with that of the Benchwork algorithm and such well-known legacy algorithms as RR, PF, EXP-PF and MLWDF. Experimental simulation results are obtained using the Vienna system level simulator [28], which is an open-source simulator released for academic and non-commercial purposes. The performance of the proposed algorithm is measured in terms of valuable metrics, such as total sector throughput, average user throughput, fairness, PDB and PLR. The simulation scenario consists of one region of interest of 10 UEs kept within the enode B2-sector1 for a fixed user scenario. The number users for the moving user scenario are varied as 5, 15, 25 and 35 to effectively conduct the analysis according to the ‘Starburst uniform walking model’. The system bandwidth used is 5 MHz spectrum, which is allocated to each sector with 500 m distance between inter enodeBs. In addition, each UE can generate two types of traffic services, namely, an H.264 video traffic with a data rate of 242 kbps and a non-GBR service with a QCI index of 7. The VoIP has a data rate of 8.4 kbps belonging to GBR service and a QCI index of 1. The simulation parameters are listed in Table 3.

**Table 3:** Simulation parameters.

Description	Value
Operating DL channel BW	5 MHz
Number of RBs	25
Active sector of concern	enodeB2-sector1
Number of active users in the targer	15, 15, 25, 35 number of users
Walking model	starburst uniform
Macroscopic path loss model	TS25814
Simulation time	50 TTIs
Speed of the user	4.16 m/s for moving user
Transmission scheme	2X2 MIMO, OLSM
Cyclic prefix used	Normal cyclic prefix
Maximum delay	0.1s

**Fig 3:**
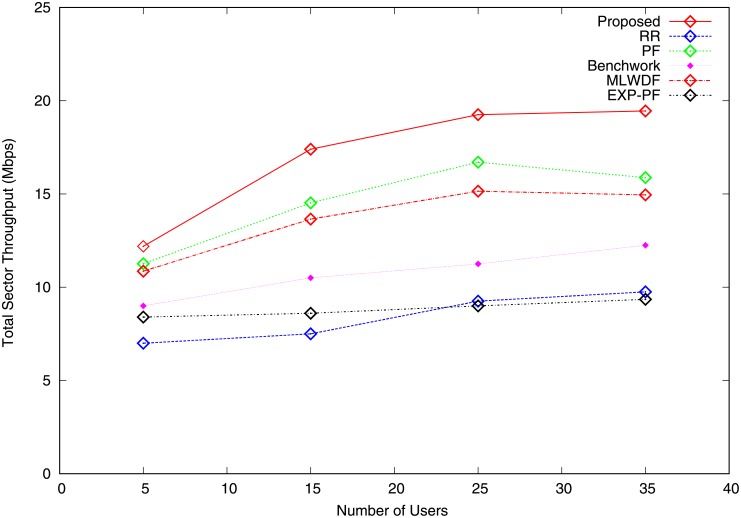
Total sector throughput video traffic.

**Fig 4:**
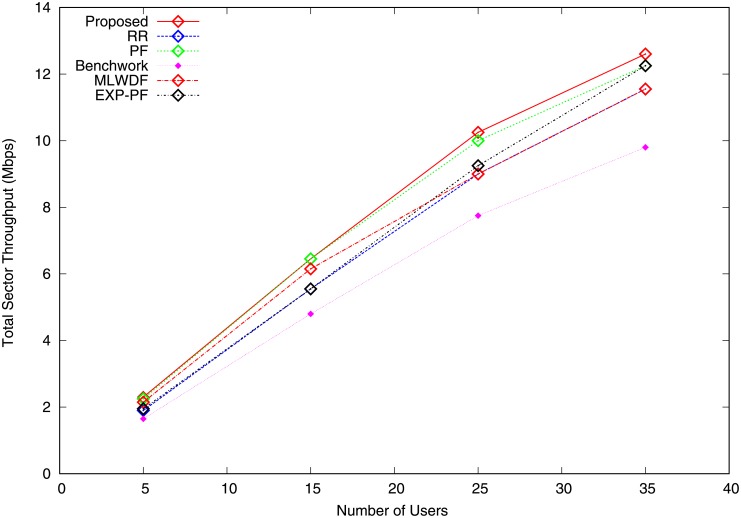
Total sector throughput VoIP traffic.

Figs 3 and 4 represent the total sector throughput for video and VoIP traffic. Fig 3 shows the numerical results for video traffic, where PF and MLWDF algorithms outperform RR, Benchwork and EXP-PF algorithms by attempting to maintain fairness between users. RR algorithm achieves the lowest total sector throughput because channel conditions are not considered in decision making. The proposed algorithm performs better than do the rest of the algorithms in terms of total sector throughput as the number of users are increased. The throughput increases because of the use of QoS class-based range to allocate RBs to bearers in good channel conditions. The proposed algorithm also performs better than do the rest of the algorithms in terms of VoIP traffic, as shown in Fig 4, because the large packet sizes are affected by different channel states. For users 5 to 18, PF algorithm has a similar pattern with the proposed algorithm but slightly goes down by varying the number of users 19 to 35. The Benchwork algorithm performs the worst of all tested algorithms, yielding the lowest throughput; good sector throughput must increase with the number of users.

**Fig 5:**
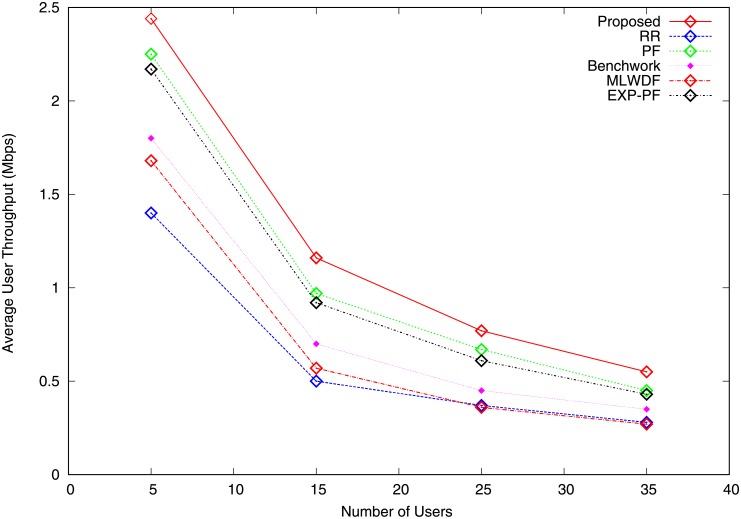
Average user throughput video traffic.

**Fig 6:**
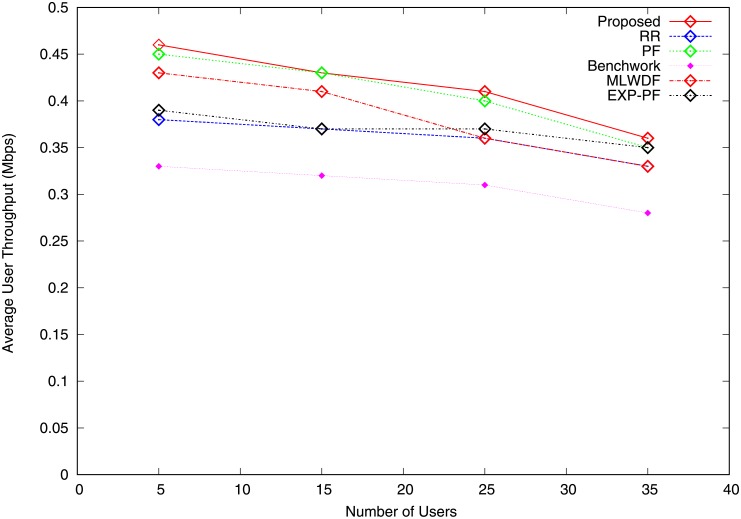
Average user throughput VoIP traffic.

The average user throughput is the ratio of the total sector throughput to the number of users. Figs 5 and 6 show the graphs of average user throughput for video and VoIP traffic. As the number of users increases, the average user throughput for all the algorithms falls. The major reason behind this behaviour is that the same quantity of RB must be shared among users. For video traffic, the proposed algorithm outperforms the others because it gives priority to GBR bearers, as shown in Fig 5. When the number of users increases from 5 to 15, the average user throughput of the proposed algorithm sharply drops, followed by a steady fall when the cell is charged with additional users because more users compete for a fixed number of radio resources. PF and EXP-PF algorithms show nearly identical throughput and are better than other strategies with the exception of the proposed algorithm. Fig 6 shows that all the algorithms show similar average user throughput for VoIP with the exception of MLWDL algorithm because they allocate resources with good channel states. MLWDF algorithm shows the worse result when the number of users is increased from 5 to 15 and becomes steady when the number of users increases from 25 to 35 because radio resources cannot be allocated in bad channel condition.

**Fig 7:**
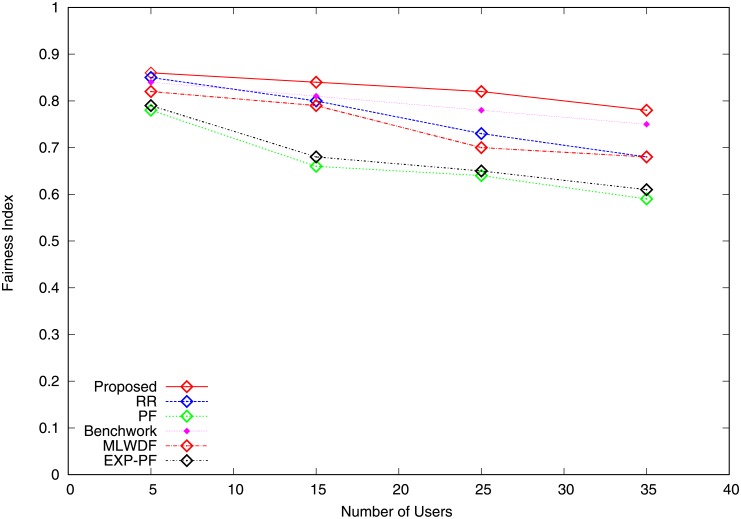
Fairness index video traffic.

**Fig 8:**
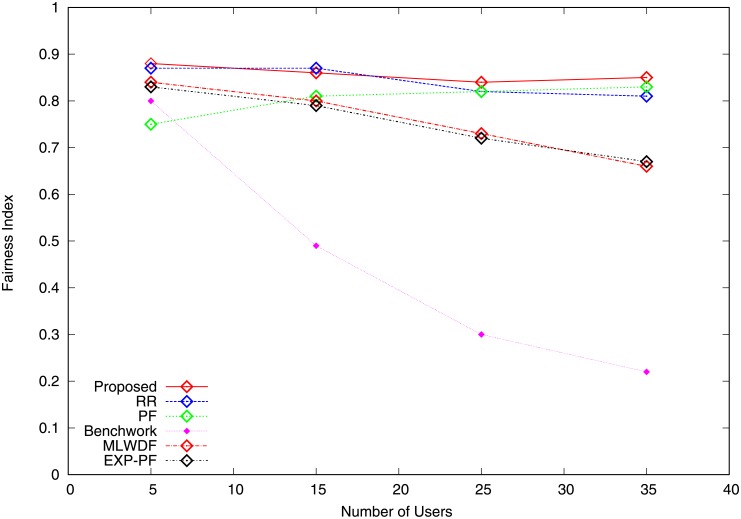
Fairness index VoIP traffic.

Figs 7 and 8 show the fairness index for video and VoIP traffic computed using the Jain fairness method [29] falls by the increasing number of users. Fig 7 shows that the fairness achieved by the proposed algorithm is the highest compared with RR and Benchwork because equal shares and fixed data rates are assigned to users. Although PF provides a trade-off between fairness and throughput, its fairness index remains poor. The fairness index for EXP-PF algorithm notably deteriorates owing to the varying number of users. The degree of fairness for MLWDF algorithm is higher compared with PF and EXP-PF algorithms, indicating that it satisfies the fairness requirement for video traffic at a certain level. Fig 8 illustrates the fairness index for VoIP traffic. The proposed algorithm shows outstanding performance in terms of fairness compared with other schemes because its capability to select users in good channel condition prevents starvation of non-GBR traffic. Benchwork algorithm shows poor fairness index; user traffic obtains better RBs share, whereas the available resources are scarce.

**Fig 9:**
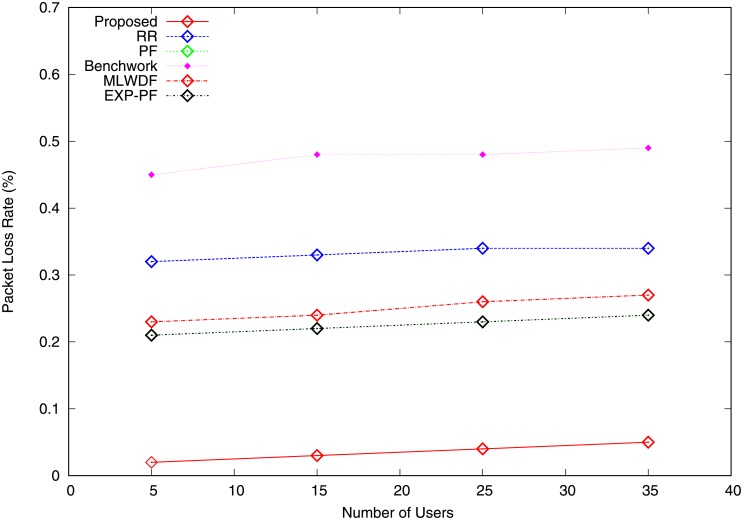
Packet loss ratio video traffic.

**Fig 10:**
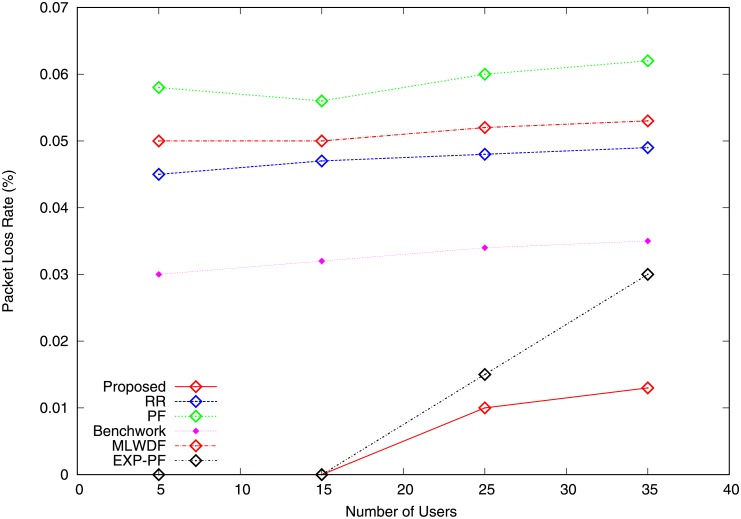
Packet loss ratio VoIP traffic.

PLR is the number of packets which fail to arrive at their destination after transmission. It is calculated as the rate of the lost packets between the total sent packets. As the number of users is increased, the PLR also increases. The PLR for video traffic of all the algorithms shows it increases with the network load, as shown in Fig 9. The proposed algorithm has a smaller PLR than do all the other algorithms because its packets reach their destination at an acceptable delay, thereby providing adequate QoS for video traffic. MLWDF and EXP-PF algorithms outperform Benchwork, PF and RR in terms of PLR because it considers packet delay in scheduling decision making. Fig 10 depicts the PLR for VoIP traffic of all the strategies. PF and MLWDF schemes for VoIP traffic drop many packets because many RBs are assigned to video traffic. Accordingly, as the number of users is increased, the VoIP traffic drops additional packets, thereby resulting in poor voice quality. The proposed algorithm reduces packet drops and results in better voice quality compared with those produced by the rest of the algorithms because small packets and highest priority are given to VoIP traffic.

**Fig 11:**
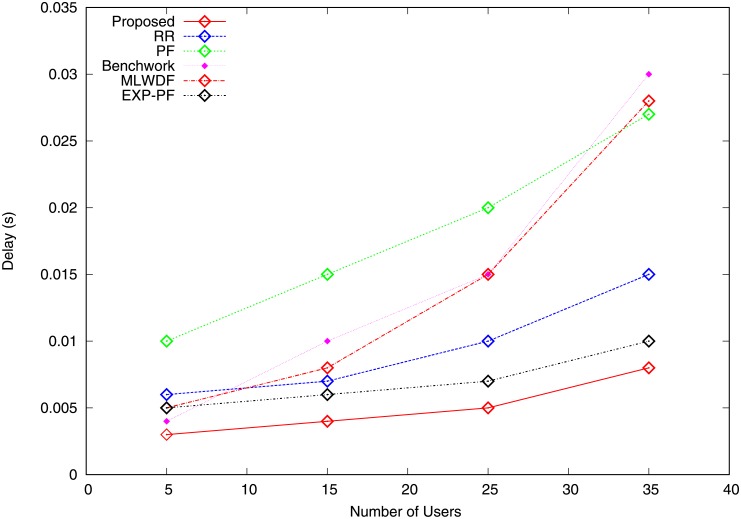
Delay video traffic.

**Fig 12:**
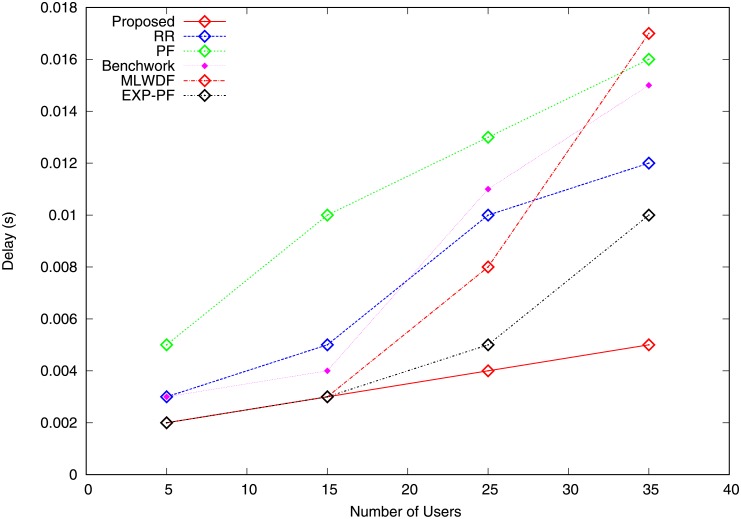
Delay VoIP traffic.

The delay is the period(s) required to transmit a packet from origin to terminal. In the network environment, if the number of users is varied, then the delay experience of the corresponding users increases as well. Figs 11 and 12 describe the delay for video and VoIP traffic, which increases by varying the number of users. Fig 11 shows that all the algorithms demonstrate an increase in delay, whereas the proposed algorithm has the least delay because its capability to allocate radio resources to the highly demanded video traffic results in the lowest PLR (Fig 9). The EXP-PF algorithm shows a small delay compared with Benchwork, RR, PF and MLWDF because it uses an exponential function of the end-to-end delivery delay in its metrics for radio resource allocation. The PF algorithm performs the worst because it tries to create fairness between traffic. Thus, it provides video traffic with many RBs and causes higher packets delays. Fig 12 illustrates the delay experienced by VoIP traffic in which the PF algorithm results in the worst delay experience compared with other algorithms because of its incapability to account for any form of delay measure. The proposed algorithm has the lowest delay because it allocates most radio resources to the largest demanded VoIP traffic and asks the video traffic to return extra RBs to enodeB to avoid waste of resources, hence resulting in smaller delay compared with other algorithms. Hence, this results in the smallest PLR compared with all the strategies illustrated in Fig 10.

**Fig 13:**
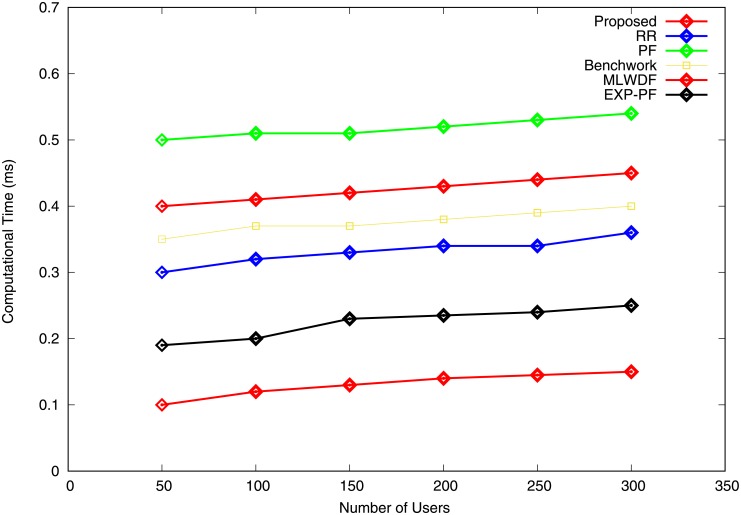
Computational time vs number of users.

Fig 13 describes the complexity analysis according to computational time for the proposed and the other algorithms. The computational time for both algorithms increases with the number of users. The proposed algorithm has 6.52% central processing unit (CPU) spent on scheduling, EXP-PF algorithm with 11.17% and 16.53% computational time is consumed by RR algorithm. Likewise, the Benchwork algorithm has 18.77%, 21.18% is for MLWDF and 25.83% CPU time is spent for scheduling by PF algorithm. Furthermore, 6.52% computational time, the minimum compared with all other algorithms, justifies that the proposed algorithm has the smallest delay and results in the lowest PLR. These advantages are explained by its capability to consider QoS requirements and user channel states before resource allocation is guaranteed.

### 4.1 Discussion

The results of our finding are discussed in this section. The results obtained in Section 4 show that the proposed algorithm, which achieves outstanding data throughput, outperforms all other algorithms when the network condition is stable because UE with better channel condition obtains more RBs. For video and VoIP traffic, all other algorithms significantly increase the PLR and the delay. By contrast, the proposed algorithm performs well by minimising the PLR and the delay at an acceptable fairness index with the help of the introduction of the new radio resource allocation scheme and considering user QoS requirements. Additionally, Benchwork, RR, PF, EXP-PF and MLWDF attempt to maintain fair transmission between traffic such that VoIP traffic can perform well. However, they still remarkably increase the average packet delay of large-demanded video traffic. Our proposed algorithm can result in better performance of VoIP with the help of bearer class priority and the resource allocation approach. The proposed algorithm guarantees the QoS requirement of video traffic with the delay tolerance of 130 ms, as shown in Table 2. Complexity analysis of computational time shows that the proposed algorithm has an outstanding performance, reduced computational time and merely 6.52% CPU time consumed for resource scheduling. These features allow the proposed algorithm to be a valid candidate for packet scheduling in future LTE technologies, such as 5G and IoT.

The proposed algorithm outperforms conventional scheduling algorithms and can thus be regarded as an innovation for downlink LTE networks.

## 5 Conclusion

In this study, we propose the QCI radio resource allocation algorithm for LTE downlinks, an improvement of the Benchwork algorithm, which lacks provision to guarantee fair transmission between traffic, thereby potentially failing to satisfy the QoS requirement of VoIP traffic and starve video traffic. To address these issues by allocating RBs, the proposed algorithm uses QCI features and different QoS parameters of traffic. In addition, the proposed algorithm compensates the need for minimum data rate by using channel states and QoS requirements, hence providing fair transmission order by allocating RBs to users in urgent need. Extensive simulation experiments are conducted to evaluate the efficiency of the proposed algorithm. The results indicate that the proposed algorithm outperforms all other schemes in terms of the metrics measured. Furthermore, it improves the total sector throughput and minimises PLR and delays for VoIP and video traffic. We aim to exploit the trade-off between resource allocation and power management between UEs in downlink LTE networks in the future.

## Supporting information

S1 File S1_File.zip. (ZIP)
